# Perspectives of Digital Health Innovations in Low- and Middle-Income Health Care Systems From South and Southeast Asia

**DOI:** 10.2196/57612

**Published:** 2024-11-25

**Authors:** Siyan Yi, Esabelle Lo Yan Yam, Kochukoshy Cheruvettolil, Eleni Linos, Anshika Gupta, Latha Palaniappan, Nitya Rajeshuni, Kiran Gopal Vaska, Kevin Schulman, Karen N Eggleston

**Affiliations:** 1 Saw Swee Hock School of Public Health National University of Singapore and National University Health System Singapore Singapore; 2 KHANA Center for Population Health Research Phnom Penh Cambodia; 3 Public Health Program Touro University California Vallejo, CA United States; 4 College of Health and Medicine Australian National University Canberra Australia; 5 Bill & Melinda Gates Foundation Seattle, WA United States; 6 Center for Digital Health School of Medicine Stanford University Stanford, CA United States; 7 National Health Authority Ministry of Health and Family Welfare New Delhi India; 8 Division of Primary Care and Population Health School of Medicine Stanford University Stanford, CA United States; 9 Department of Pediatrics School of Medicine Stanford University Stanford, CA United States; 10 Clinical Excellence Research Center School of Medicine Stanford University Stanford, CA United States; 11 Shorenstein Asia-Pacific Research Center Freeman Spogli Institute for International Studies Stanford University Stanford, CA United States

**Keywords:** digital health innovations, public health, South and Southeast Asia, health care challenges, low- and middle-income countries, LMICs, global health, health AI, artificial intelligence, public health responses, global health contexts, digital health

## Abstract

Digital health innovations have emerged globally as a transformative force for addressing health system challenges, particularly in resource-constrained settings. The COVID-19 pandemic underscored the critical importance of these innovations for enhancing public health. In South and Southeast Asia, a region known for its cultural diversity and complex health care landscape, digital health innovations present a dynamic interplay of challenges and opportunities. We advocate for ongoing research built into system development and an evidence-based strategy focusing on designing and scaling national digital health infrastructures combined with a vibrant ecosystem or “marketplace” of local experiments generating shared experience about what works in which settings. As the global digital health revolution unfolds, the perspectives drawn from South and Southeast Asia—including the importance of local partnerships—may provide valuable insights for shaping future strategies and informing similar initiatives in low- and middle-income countries, contributing to effective digital health strategies across diverse global health contexts.

## Introduction

The rapid development of digital health—encompassing technologies like mobile health (mHealth), telemedicine, digital health information systems, Internet of Things, machine learning and artificial intelligence (AI), personalized digital nudges, large language models, and generative AI—has ushered in a new era of health care possibilities [1]. Digital health technology has been proposed as an effective solution to address health care access, cost, and quality issues. While the promise of digital health is universal, the context in which it is applied varies across communities and regions. This viewpoint paper critically examines the diverse challenges and opportunities encountered in digital health innovations in low- and middle-income countries (LMICs), drawing on perspectives from the experiences of health systems in South and Southeast Asia, a vibrant and multifarious region.

Just as many have highlighted the advantages of a “human-in-the-loop” approach to AI, we emphasize the potential benefits of a “researcher-in-the-loop” approach to digital health innovations in LMICs. This approach aims to ensure objective evaluation, continuous improvement, and effective dissemination of successes and failures, ultimately enhancing patient outcomes while reducing disparities, costs, and the burden and burnout of health professionals. A trilateral team comprising policy makers, researchers, and development partners can expedite the identification and dissemination of best practices across different LMIC settings. Additionally, partnering with trusted global organizations or catalytic funders—such as the Bill & Melinda Gates Foundation (BMGF), National Institutes of Health Fogarty International Center, Abdul Latif Jameel Poverty Action Lab, and the International Initiative for Impact Evaluation—can provide essential resources to low-resource settings, mitigate risks during the initial adoption phase, and support adapting interventions to the local context before scaling up to larger populations. This collaboration also helps build a regional or global “toolkit” of use cases and experiences that can benefit other LMICs [2].

The following sections will first articulate our analytic framework and examine the foundational elements of digital health innovations, focusing on their architecture, standards, and issues related to privacy and security. Next, we will explore the digital health solutions marketplace in South and Southeast Asia, emphasizing the importance of local community partnerships and the need for a continuous cycle of learning and improvement through impact evaluation, research, and development. We emphasize the need for cultural sensitivity and prioritizing equity to ensure that digital health solutions are effectively adapted to local contexts.

### Improving the Social Net Benefit From Digital Health Innovation: Analytic Framework

Our analytic framework considers balancing the social benefits of digital health innovations with the social costs of adoption, recognizing (1) the positive spillovers from evidence generation and (2) the opportunity costs of not adopting innovations that might otherwise have improved health and equity at affordable cost. Critically, the social benefits should consider the incremental global public good of collecting evidence and experience about what works in diverse local settings. If these spillover benefits for other LMICs are ignored, resource constraints will lead to underinvestment in digital health innovation for low-resource communities. To leverage additional resources to align with social benefit (to internalize this externality, in economics jargon), partners can support local experimentation and evidence generation. Regional or global partners can help leverage existing technology platforms and standards to generate economies of scale and global public goods. Cutting-edge applications can be tailored and redeployed in multiple low-resource settings, allowing LMICs to decrease implementation costs, foster the development of reusable tools and methodologies, and nurture culturally relevant communities of practice.

To balance social benefit and cost while taking account of these spillovers, ongoing research should be built into system development. We advocate for a 2-pronged top-down and bottom-up innovation approach: an evidence-based strategy focusing on designing and scaling national digital health infrastructures combined with a vibrant ecosystem or “marketplace” of local experiments generating shared experience. Importantly, evidence about failures and successes is essential to reduce the costs of deploying ineffective strategies.

This dual approach of top-down design and bottom-up experimentation, enabled through careful evaluation of effectiveness and impact, allows economies with different starting conditions to seize opportunities to “leapfrog” toward more robust, resilient health systems fitting their contexts rather than imitate the development path of any given current high-income country or region. This theoretical framework emphasizes that the incremental social net benefit of digital innovations depends critically upon the “starting point” and desired “end point”—both of which rely on local context and require local partner input to accurately assess benefits and costs.

For example, the starting point for any “leapfrog” in technology may differ in light of significant technological disparities—“the digital divide.” Despite the rapidly advancing technological landscape in LMICs, substantial disparities in digital access and digital literacy persist. Across South and Southeast Asia, less than half of the population in Myanmar uses the internet, and only 2% of people in Cambodia and the Lao People’s Democratic Republic have a fixed broadband subscription compared to an average of 4% in LMICs [3]. Cambodia has low digital literacy [4], whereas in countries like Singapore, digital literacy and technology use are relatively high, even among older adults [5]. Digital literacy can impact one’s ability and attitude toward using digital technology [5,6]. The scarcity of technological resources in LMICs can also hamper the introduction, adoption, and integration of digital health interventions into the broader health system and compound existing inequities in access to health care, potentially exacerbating disparities in health outcomes [7,8].

Yet, we emphasize that the incremental benefits of digital technologies could be significant—even transformational—in LMICs. By providing access to quality care in remote settings, the incremental benefits could be considerably higher than in high-income health systems if sufficient oversight and stewardship uphold the tenet “First, do no harm.” The ethical complications of denying access to the social value of digital health innovations cannot be ignored.

Local partnerships can reduce the social costs of developing appropriate digital technologies and enhance their social benefits by creating trust for engagement and evidence aligned with local community goals. To emphasize the net benefit of understanding existing traditions or policies before attempting to change them, we use the concept of “Chesterton’s Fence” based on the writer Chesterton [9]: “If you come across a fence blocking a road and do not know why it was built, you should not remove it until you understand its purpose.” Digital health innovations should not blindly “leapfrog” Chesterton’s Fence: reforms or changes should not be made until the reasons for the current situation are fully understood. Incorporating historical knowledge and avoiding hasty or uninformed decision-making can enhance the net social value of changes.

For balancing the social benefits and costs of digital health innovation, Chesterton’s Fence suggests the importance of actively involving local communities, respecting local decision-making processes, and partnering with local governments, nongovernmental organizations (NGOs), and other stakeholders to tailor proposed solutions to the cultural, economic, and environmental context. Implementing small-scale pilot projects can provide valuable insights, allowing for adjustments before broader application, considering both the direct and indirect consequences for the community. Moreover, the social benefits and costs should be measured over a forward-looking time frame, not limited to short-term results. Sustainable change often requires investments in local skills and institutions to ensure long-term effectiveness. Patience and persistence are vital in these efforts to harness the potential of digital technology to improve the health of disadvantaged individuals and communities. Ongoing evaluation and willingness to adapt strategies based on feedback are critical for maximizing the social net benefit of digital health innovations.

### Data Architecture, Standards, Privacy, and Security

For digital health technology to have a long-lasting impact on health systems, technology must be developed around an extensible architecture that can be efficiently scaled for new use cases and apps. While we are at the very early stages of technology adoption, it is essential to understand how critical these foundational concepts are to a proper digital solution for health care. An essential aspect of this adaptable architecture is incorporating data governance to facilitate the development of use cases and technology deployment.

Electronic medical records have been used to streamline patient data management and improve health care service delivery, exemplifying digital health’s potential to improve health care efficiency [10]. The Indonesian and Singaporean governments are making electronic medical records accessible to the population [11,12], while an electronic information registry for routine immunization is being introduced in Cambodia and the Lao People’s Democratic Republic. However, with specific data standards for organization and sharing, data elements may be digitally exchanged between clinical systems.

The process of exchanging digital information is known as interoperability. Most high-income countries have either implemented or are in the process of introducing legislation mandating interoperability [13]. However, many legacy health information technology solutions are designed primarily for providers, hospitals, or clinics, which supports organizational goals but presents challenges for data sharing and exchange as patients move across different providers. In contrast, only some LMICs have achieved comparable levels of interoperability. Nevertheless, LMICs may have the most promising opportunities to “leapfrog” older standards by directly adopting a patient-centered data architecture.

Rather than developing a data architecture that mirrors the health ministry organizational chart, policy makers should consider technology solutions centered on patients, such as personal health records. In Singapore, the government has launched the Next Generation Electronic Medical Record system to consolidate data and harmonize processes across health establishments, including private care providers, providing a longitudinal view of patient’s health data [14]. Such efforts to integrate digital health solutions into existing health care frameworks hold promise in optimizing resource allocation and health care delivery. Mobile phones have greatly facilitated personal health records but could include cloud-based models or an innovative blockchain model such as that used in Estonia [15]. Personal health records could have distinct advantages for patients, such as having patient records available when making clinical decisions.

**Figure 1:**
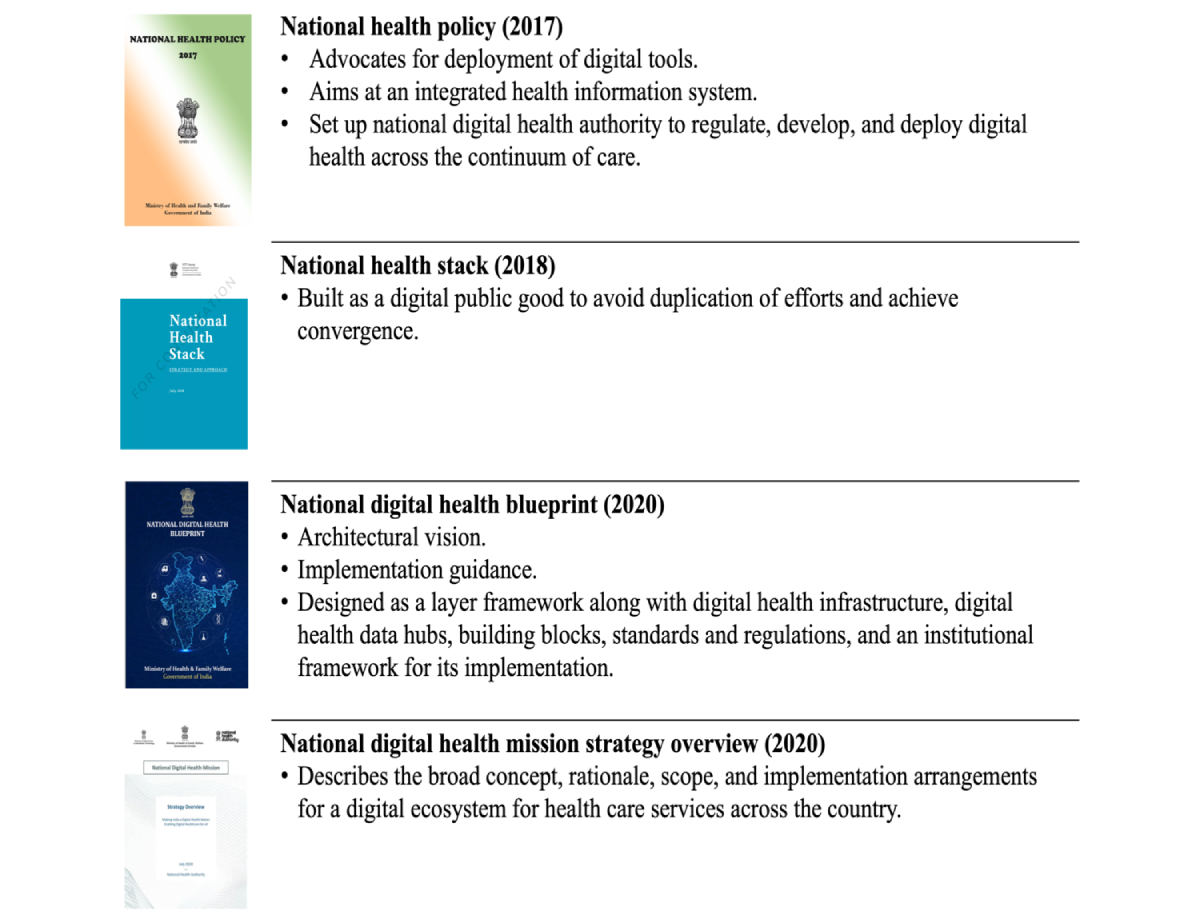
India’s digitalization policies, strategies, and experiences [16-19].

Launched in 2021, India’s national Ayushman Bharat Digital Mission (ABDM) was built based on a decade of digitalization experiences in the national identity system, personal digital records, and payment systems (Figure 1[16-19]). ABDM uses the Ayushman Bharat Health Account as a digital health identity number that can be shared freely while keeping the underlying information secure. Over 268,000 health professionals have been included in the national registry, irrespective of the medical systems they practice. More than 229,000 health facilities have been registered with the facility registry [20].

A key lesson from India’s experiences with ABDM is building an integrated data ecosystem instead of disparate information systems. The integration will be crucial to support personalized health care and evidence-based policy making. The overall data architecture at a country or market level should also anticipate the interest in developing these tools and their challenges. Individual tools should anticipate updates to the overall data architecture and regulation at the country and market level.

Apart from data architecture for storage and exchange, data standards vary across countries, leading to incomparability [21]. The same concept (eg, blood glucose) may be represented in various ways from one setting to the next. The World Health Organization has developed a data architecture to support countries’ capacity to collect, manage, analyze, and use health data from population-based sources such as household surveys and institutional sources such as health facilities [22]. Similarly, Digital Health Europe attempts to develop comprehensive data standards, including international patient summaries that provide primary medical data for supporting cross-border care [23]. The European case of regional harmonization is a model for regional organizations such as the Association of Southeast Asian Nations, a political and economic union of 10 Southeast Asian states, to work toward fostering data exchange and improving data comparability between countries.

**Figure 2:**
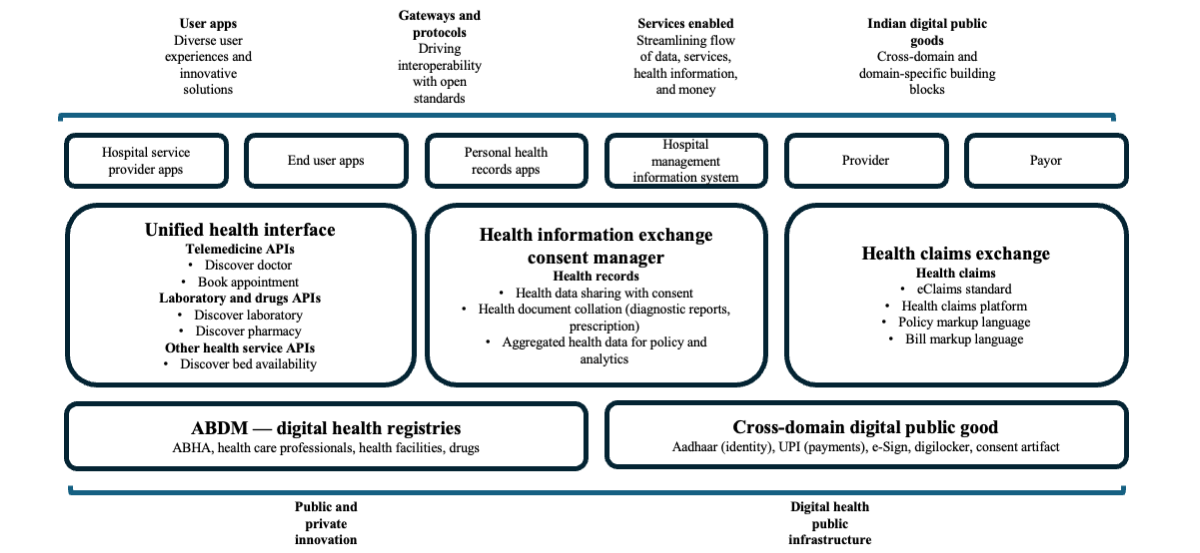
India’s ABDM data architectural elements and underpinning services and features. ABDM: Ayushman Bharat Digital Mission; ABHA: Ayushman Bharat Health Account; API: application programming interface; UPI: Unified Payments Interface.

Finally, moving from paper to digital records raises the risk of data breaches and unauthorized access to sensitive health data, which poses ethical and legal dilemmas. Some countries have tightened regulations to increase protection. Singapore’s Personal Data Protection Act is a regulatory model for safeguarding personal health data [24]. In Thailand, Indonesia, and Vietnam, data protection was embedded in several laws before its personal protection law was passed in 2019, 2022, and 2023, respectively [25-27]. India has adopted the Digital Personal Data Protection Act, which empowers individuals and specifies requirements for any data fiduciary involved in handling personal data, as well as for the National Health Authority, which serves as a consent manager rather than a data repository [28]. India’s ABDM also includes a consent artifact that empowers users to decide how, when, where, and what record types they allow to be shared between one health provider and another (Figure 2).

However, inadequate data protection regulations in some countries risk the security and privacy of health data [29], which could undermine individuals’ willingness to engage with digital health platforms. An example is the apprehension surrounding Indonesia’s new Health Bill, which allows for collecting and using health data, including genomic data, outside the country [30]. Clear guidelines for data collection, storage, and sharing, along with measures to safeguard sensitive health information, are critical.

These architectural elements are designed for 3 primary functions: interoperability of health records, interoperability of services through the unified health interface for all providers, and interoperability of health claims so that hospitals or other providers are manageable when dealing with multiple payers to submit claims. Together, these layers create a citizen-centric view of public health by building the infrastructure underpinning a vibrant ecosystem. Featuring 3 main exchanges—the Unified Health Interface, the Health Information Exchange consent manager, and the Health Claims Exchange—this system has succeeded in integrating more than 1280 different public and private apps and laying the foundation of digital health public infrastructure for ongoing public and private innovation (Figure 2). India continues to explore strategies to drive further adoption and social value.

### Marketplace—Digital Health Solutions

The genuine excitement surrounding digital health care solutions stems not from data architecture but from the services they enable. Data architectures are not a final use case for a technology; they are merely an enabler of a broader digital health care services ecosystem. Digital services use an underlying data architecture to provide patient information, tools, reports, and recommendations. Digital services can be built on a standard data architecture or stand-alone solutions. The former requires a means to access and potentially update or add to the data, while the latter runs the risk of creating noninteroperable data silos. We advocate for adopting both approaches while systematically assessing the latter and designing in anticipation of interoperable solutions in the near future. Digital solutions must be tested to ensure they perform as expected, including concepts such as the user interface; data access, storage, manipulation, and reporting; and clinical validity.

A vibrant ecosystem of apps shown to be effective in different settings, in turn, lays the foundation for a “marketing strategy” to disseminate and encourage the take-up of digital health solutions such as digital health records among individuals and organizations. Building the personal health report system means little if patients and providers do not engage. To drive meaningful adoption and use, the digital health ecosystem must create value for every player or stakeholder, fostering trust and spurring curiosity about the possibilities for more manageable, cheaper, higher quality, more accessible, and more resilient services. The patients and providers need evidence from use cases that improve their lives, transforming inertia and fear of new technology into excitement and fear of missing out. Digital solutions must also easily integrate into existing workflows and systems, reducing the barriers to uptake.

For example, the Indian government started by ensuring that all the government programs, such as ABDM-enabled health and laboratory management information systems for government hospitals, are ABDM-enabled. Such deployment creates the demonstration space to study integration into existing workflows and make these low-cost “plug and play” solutions available to all participants so that even the smallest local provider need not invest much time and money to adopt them.

While the goal is to avoid creating siloed platforms—an HIV platform, a TB platform, and so on—as much as possible, health systems may benefit from a vibrant ecosystem or “marketplace” of local apps and experiments generating shared experience about what works in diverse settings to enhance access while reducing disparities. One noteworthy example from Southeast Asia comes from a recent randomized controlled trial that demonstrated the benefits of digital health interventions for accessing health care and support services among female entertainment workers, a social and gender-marginalized population in Cambodia [31]. The study used a mobile intervention to connect the vulnerable population to critical health and gender-based violence services, showcasing the transformative potential of digital health in addressing pressing societal challenges even without a national-level integrated digital health ecosystem.

The potential of digital health to bridge geographical gaps, extend health care services to underserved populations, and support the health system in moving care from hospitals to communities is promising. In archipelagic countries like Indonesia and the Philippines, as well as in countries such as Cambodia, where 75% of the population lives in rural areas [3], digital health interventions such as teleconsultation can be used to improve health care accessibility. Digital health additionally holds potential in countries reliant on community health workers to bridge geographic gaps. For example, Accredited Social Health Activist workers in India, who are employed by the Ministry of Health and Family Welfare through India’s National Rural Health Mission, have successfully leveraged mHealth for disease surveillance and treatment in a country where low-cost smartphones are increasingly prevalent [32,33].

A mobile phone can be considered a valid medical device equipped with multiple sensors: a camera, a microphone, a screen, a global positioning system, and data. Organizations like BMGF are working with local partners to develop apps leveraging the ubiquity of mobile phones to enhance care in low-income settings, such as scanning to assess a newborn’s anthropometrics and monitor malnourishment. mHealth has been proven effective to varying degrees in health education, disease screening, and linkages to care and treatment services [34-38], while wearable devices can increase physical activity and improve health [39], and Internet of Things–enabled systems can support community eldercare [40]. An app for counseling women about contraceptive methods showed that for the informed adoption of unfamiliar technologies, personalized information could be just as effective and far cheaper than subsidies [41]. The power of social media as a platform for health education also holds considerable promise both for large-scale public health campaigns and specifically tailored interventions that are culturally sensitive and build on trusted community figures or institutions [42].

AI has demonstrated significant potential in supporting precision health, including the use of large language models and generative AI for diagnosing complex medical conditions [43]. For example, integrating a deep learning algorithm into Thailand’s national diabetic retinopathy screening program has shown that this system can provide real-time detection of diabetic retinopathy with accuracy comparable to that of retina specialists in community-based settings [44]. AI-enabled solutions for precision public health extend beyond diagnostics to clinical decision support and health care delivery, particularly in low-resource settings where staffing, training, funding, and equipment are often limited. While AI solutions may rely on effective collaboration with academic and industry partners, NGOs can also play a crucial role in data acquisition and technology adoption, as detailed in the following section [45].

Ultimately, digital health solutions aim to augment, not replace, the services provided by frontline health workers—we need to avoid the “Turing Trap” [46] and focus on innovations that augment the social value created by the existing health workforce. In the most recent and rigorous studies, generative AI and related technologies tend to boost the productivity of new and less-experienced health workers more than their more-experienced counterparts [47]. The potential for AI and other digital health assistants to upskill and extend the limited health professional resources of LMICs underscores the social value of experimenting and assessing real-time effectiveness without waiting for the design of national architecture where it is lacking.

Digital health innovation also needs to consider the financing levers in both the public and private sectors to understand the feasibility of adopting and scaling up digital health interventions. A private philanthropy like BMGF can provide catalytic funding to derisk the technology early and gather evidence about what works in practice before turning over implementation at scale to governments and civil society. For example, BMGF successfully helped deploy a platform developed in India in 2 other countries, Cambodia and Nigeria [48,49]. Using technologies to support health system strengthening through health financing also holds promise for increasing health care efficiency, accountability, and transparency [50]. Digital technologies have been used to collect health insurance premiums and taxes through mobile wallet services, support pay-for-performance strategic purchasing models, and raise awareness of benefit packages and cost schedules [50].

### Local Partnerships for Successful Digital Health Implementation

The architecture on which digital technologies are built may be universal, but the services they provide must be localized to address local needs and be sufficiently engaging to promote adoption. One example of the importance of tailoring digital health innovations to the local context comes from maternal and child health in India [51]. Many Indians, especially in rural areas, are not born in their parents’ houses or even their parents’ towns but rather in their maternal grandparents’ houses or communities. Typically, in the third trimester of pregnancy, the pregnant woman will move to her parents’ house, and the baby will be born there. Since the third trimester is a critical time for the mother and child, customized software supported by the BMGF alerts community workers to gather 2 addresses for each pregnant woman and then sends an alert to the maternal grandparents’ community in the third trimester. This allows for follow-up care and management continuity, ensuring that health care workers in each jurisdiction are aware of incoming cases, thereby reducing the number of pregnant women lost to follow-up at this critical time.

Local champions and partnerships are critical in providing cultural nuances, context, and advocacy, and in some instances, in creating a network of users and customers for realizing the full social benefit of digital technologies. NGO Arogya World led one example of local champions and partnerships enabling successful digital health implementation in India. Arogya World has developed and implemented several diabetes prevention programs that have successfully incorporated digital tools. Their program mDiabetes, a text messaging intervention delivering diabetes education, significantly improved health behaviors and nutrition [52]. The Healthy Schools Program, a school-based intervention targeting 11- to 14-year-olds, incorporated digital curricula in light of the COVID-19 pandemic. Healthy Schools Program achieved a broader reach through digital campaigns, yielding notable gains in knowledge about diabetes, its risk factors, and behaviors spanning nutritious food consumption and physical activity.

Another example comes from the NGO Noora Health, which has successfully leveraged digital health across India, Bangladesh, and Indonesia to improve access to caregiver training using multimedia. Successful features of their programs include mobile chat services and digital curricula integrated into comprehensive care delivery models that use local health care systems. Through meaningful technology adoption and partnerships with local governments, policy makers, and community health workers, NGOs like Arogya World and Noora Health are well-poised to launch large-scale public health campaigns that cater to increasingly technology-savvy populations.

Local partnerships are crucial in successfully implementing community-based digital health interventions, as evidenced by the Mobile Link and i-MoMCARE projects in Cambodia. Led by the Khmer HIV/AIDS NGO Alliance, a leading local NGO, the Mobile Link used a participatory approach to involve representatives of the female entertainment worker community, civil society organizations, and policy makers in formative studies and intervention development, implementation, and evaluation [31]. This partnership with local stakeholders has significantly increased access to services and improved health outcomes in the target population. Similarly, i-MoMCARE leverages primary health care providers, community health workers (known as village health support groups in Cambodia), a local university, and the national maternal and child health program to provide maternal and child health education and reminders via a mobile app [49]. This enables real-time data collection and personalized care plans, ensuring timely antenatal, delivery, and postnatal care for mothers and children in rural Cambodia. These partnerships facilitate trust, cultural relevance, and sustainability, ultimately enhancing the effectiveness and reach of digital health technologies. Additionally, the involvement of trusted and reliable local sources of information improves the credibility and acceptance of these digital health interventions. A rigorous impact evaluation is part of the design for i-MoMCARE so that this experience can contribute evidence sound for other low-resource settings globally.

Local champions and advocates provide the social legitimacy necessary for the adoption and effective use of digital technologies for health and well-being. This is particularly important in the realm of AI, where apps still need to be thoroughly tested at the population level and where security breaches have undermined public confidence. In some contexts, these technologies could even be seen as tools for surveillance and propaganda. Social legitimacy thus seeds the trust required for the adoption and effective use of the technology. For example, a large-scale randomized controlled trial in West Bengal, India, found that SMS text messages from West Bengal native and 2019 Nobel laureate Abhijit Banerjee doubled the reporting of health symptoms to community health workers and had other positive spillover impacts on their communities [53]. This example illustrates the power of communication technology and potentially social media as platforms for health education and public health information for large-scale campaigns and specifically tailored interventions that build on trusted community figures or institutions.

### Digital Health Innovations—A Cycle of Learning and Improvement

The rapid proliferation of digital health technologies requires rigorous research and impact evaluation to generate the evidence to assist LMICs in identifying the most effective strategies. Mobile phones and their voice and AI apps provide many opportunities to enhance individual health knowledge and deliver helpful information. They also call for careful ongoing research to address inappropriate advice or information concerns. For example, standard smartphone voice systems (eg, Siri or S Voice) have failed to recognize issues about mental health, interpersonal violence, or physical health [54]. The accuracy of AI-generated information about diabetes is also questionable [55]. Many ethical issues need to be addressed when adopting monitoring devices or contactless sensors and contact-based wearable devices installed in health care settings, otherwise called “ambient intelligence,” to collect data in health and elderly care [56]. AI or machine learning also relies on models that require significant amounts of data for development and attention to the potential errors introduced by data that may be flawed or biased [57]. Validation of the models across health care markets and populations is essential [58].

Digital health research requires an interdisciplinary team of researchers in public health, data science, information technology, social sciences, and engineering. Researchers must work with policy makers and end users such as health care professionals, communities, and individuals in designing interventions that are problem- or need-driven and set in the broader policy environment. The principle of equity must guide the innovation cycle to prevent inadvertently deepening health disparities through underdiagnosis and undertreatment of individuals historically with limited access to care or who face biases due to language, race, or education. For example, using medical spending to predict medical needs can result in significant racial bias [59]. Strategies to reach marginalized populations, including migrants and underserved communities, are imperative. Initiatives such as using mobile phones to deliver health information to key HIV population communities in Cambodia reflect a commitment to advancing health equity [31,60].

In addition, ensuring that participants comprehensively understand the implications of digital health research, especially in linguistically and culturally diverse contexts, is paramount. Countries in the region have taken steps to develop culturally sensitive informed consent processes to uphold ethical standards and respect individuals’ autonomy [30]. The digitization experience of the banking and financial industry in the region can also offer valuable lessons for digital health research and interventions. The banking sector has learned that retaining a human touch in the technological interface with customers is essential [34]. In some countries where public health services have been underused due to the lack of trust in the system, the introduction of digital health interventions must be carefully designed to instill confidence in the intent, quality, and integrity of the technology [35]. Retaining a human element in digital health interventions could be one way to instill confidence.

The key to success will be alacrity in the stewardship of health sectors to address priority population health needs and improve equity. Policies should support the generation of evidence assessing digital health apps so that patients, providers, and policy makers can ask and answer the right questions in a suitable time frame to enable a virtuous cycle of learning and improvement. In LMICs, where governments lack resources to try many new things, private philanthropy can bring resources, take the risk, and generate evidence about “what works” and scalability.

While some may perceive the term “leapfrogging” as imposing a development paradigm that prioritizes Western values and homogenizes diverse cultures, we suggest that each culture and economy may choose its leapfrog developmental path tailored to its unique circumstances. Although space limitations preclude a full exploration of alternative development models within this brief perspective piece, future research should prioritize an in-depth discussion of related theories and evidence. This includes conducting cost-benefit analyses of heterogeneous digital health interventions across LMIC contexts.

### Conclusions

Digital health programs and research in South and Southeast Asia illustrate that innovation can deliver a social net benefit to communities while generating positive spillovers for other LMICs based on a comprehensive understanding of technological, cultural, and ethical dimensions of the intervention and community context. While technological challenges persist, opportunities for supplementing traditional health care, strengthening health systems, and enhancing epidemic preparedness hold promise. At the current unprecedented time in humanity’s digital transformation, these discussions are topical and urgent. Ethical considerations underscore the importance of equitable access, cultural sensitivity, robust data governance, and partnering with local communities. Collaborative efforts among researchers, policy makers, health care professionals, and civic organizations or other community representatives within and outside the health sector will be instrumental in harnessing digital health’s potential to improve health outcomes and promote health equity in LMICs.

## Abbreviations

ABDM: Ayushman Bharat Digital Mission
AI: artificial intelligence
BMGF: Bill & Melinda Gates Foundation
LMIC: low- and middle-income country
mHealth: mobile health
NGO: nongovernmental organization
